# Associations and Additive Interaction of Childhood Trauma and Psychological Resilience With Depression Among Vocational College Students

**DOI:** 10.62641/aep.2199

**Published:** 2026-10-15

**Authors:** Jiacun Tian, Xiaoping Chen, Danwen Huang, Xiaoyan Qiu

**Affiliations:** ^1^Student Affairs Office, Yangming School of Shaoxing Vocational & Technical College, 312000 Shaoxing, Zhejiang, China; ^2^Academic Affairs Office, Medical School Shaoxing Vocational & Technical College, 321000 Shaoxing, Zhejiang, China; ^3^Mental Health Education Guidance Center, Zhejiang Post and Telecommunications College, 321000 Shaoxing, Zhejiang, China

**Keywords:** childhood trauma, psychological resilience, vocational college students, depressive symptoms, interaction

## Abstract

**Background:**

To observe the effects of childhood trauma and psychological resilience on depression in vocational college students, as well as their additive and interactive effects.

**Methods:**

From November 2023 to November 2024, 992 college students from a higher vocational and technical college in Shaoxing were selected using cluster sampling. An online survey was conducted using the Center for Epidemiologic Studies Depression Scale (CES-D), the 10-item Connor-Davidson Resilience Scale (CD-RISC-10), and the Childhood Trauma Questionnaire-Short Form (CTQ-SF). According to the presence or absence of depressive symptoms, the students were divided into a depressive symptom group and a non-depressive group. CTQ-SF scores, CD-RISC-10 scores, and general characteristics of were compared between the two groups to analyse factors associated with depressive symptoms among higher vocational college students. The additive interaction between CTQ-SF and CD-RISC-10 scores in relation to depressive symptoms was also analysed.

**Results:**

Among the 992 vocational college students, the incidence of depressive symptoms was 19.86% (197/992). The CTQ-SF score was higher in the depressive symptom group than in the non-depressive group (*p* < 0.05), and the CD-RISC-10 score was lower in the depressive symptom group than in the non-depressive group (*p* < 0.05). The depressive symptom group was older than the non-depressive symptom group and had higher proportions of students with monthly living expenses <1000 yuan, average or poor relationships with classmates, and divorced or widowed parents (*p* < 0.05). Multivariable logistic regression analysis showed that age (odds ratio (OR) = 1.179, 95% confidence interval (CI): 1.072–1.295), average or poor relationships with classmates (OR = 3.197, 95% CI: 2.038–5.015), and having divorced or widowed parents (OR = 3.208, 95% CI: 1.788–5.756) were risk factors for depressive symptoms among vocational college students (*p* < 0.05). Monthly living expenses (OR = 0.264, 95% CI: 0.168–0.416), sexual orientation (OR = 0.740, 95% CI: 0.551–0.993), and CD-RISC-10 score (OR = 0.306, 95% CI: 0.187–0.502) were protective factors for depression symptoms among vocational college students (*p* < 0.05). The additive interaction analysis yielded relative excess risk due to interaction (RERI) = –0.001, attributable proportion due to interaction (AP) = –0.002, and synergy index (S) = 1.003, indicating no additive interaction between CTQ-SF and CD-RISC-10 scores in relation to depressive symptoms among vocational college students.

**Conclusions:**

CTQ-SF and CD-RISC-10 scores showed no additive interaction in relation to depressive symptoms among vocational college students.

## Introduction

Depression is a common and complex mental disorder, mainly characterised by significant and persistent low mood. Patients may exhibit extreme behaviours such as self-harm or suicide [1]. Among college students, depression may be associated with a variety of factors, including difficulties adapting to a new environment, academic pressure, interpersonal and romantic problems, financial stress, and uncertainty in the job market. These factors may interact and collectively affect students’ mental health, thereby increasing incidence of depression [2, 3]. Vocational college students constitute a distinct group in higher education. Their education is skill-oriented, and their academic pressure differs from that of students in traditional colleges and universities. Their assessment methods are relatively diverse, and some students may experience anxiety and self-denial due to a weaker academic foundation [4]. Moreover, vocational college students are more likely to enter industries characterised by high work intensity and limited opportunities for career advancement after graduation, such as manufacturing and service industries, which may further aggravate their psychological burden [5].

Childhood trauma refers to adverse life events experienced during childhood that exceed an individual’s coping abilities, such as domestic violence and parental neglect. Such experiences can have profound effects on their physical and mental health [6]. A previous study showed that individuals who experienced childhood trauma were more susceptible to depression in adulthood; however, not all such individuals will develop depressive symptoms [7]. Some individuals have a stronger psychological resistance to external pressure and therefore exhibit a lower incidence of depressive symptoms despite exposure to childhood trauma [8].

Psychological resilience refers to an individual’s ability to recover and adapt quickly when faced with adversity, pressure, setbacks or trauma. Psychological resilience is a plastic and dynamic capacity, and its regulatory effect can reduce the suicide rate [9]. At present, few studies have examined the relationships and interactions among childhood trauma, psychological resilience and depressive symptoms vocational college students.

Therefore, this study used random cluster sampling to investigate the current status of depressive symptoms, childhood trauma and psychological resilience among vocational college students. The findings may provide theoretical reference for the prevention of depressive symptoms in vocational college students.

## Materials and Methods

### Subjects

From November 2023 to November 2024 (excluding examination periods and holidays), a questionnaire survey was conducted among students of a higher vocational and technical college in Shaoxing using random cluster sampling.

Inclusion criteria: (1) Students of a higher and technical vocational college in Shaoxing, Zhejiang Province; (2) Age ≥18 years old; (3) Informed consent and voluntarily participated in this study. Exclusion criteria: (1) History of a diagnosed mental disorder; (2) Major life events within the previous 6 months, such as death of a relative or a serious accident; and (3) incomplete questionnaire or responses showing obvious response patterns. A total of 992 eligible students from the selected classes participated in the survey.

Before the survey, the class teacher or counsellor explained the purpose of the study and the students participated in the online questionnaire after giving informed consent. The online questionnaire was distributed by the counsellor or class teacher to students in the randomly selected classes. A total of 1015 questionnaires were distributed, of which 992 were valid, yielding an effective response rate of 97.73%. The study included 415 males and 577 females, with a mean age of 19.15 ± 2.58 years.

This study was approved by the Ethics Committee of Yangming School of Shaoxing Vocational & Technical College (institution review board number, 20231220) and was performed in accordance with the principles of the Declaration of Helsinki. Informed consent was obtained from all individual participants included in the study.

### Methods

#### General Characteristics

General characteristics were assessed using a self-developed questionnaire covering age, only-child status, gender, sexual orientation, place of residence, monthly living expenses, daily internet use, parental education level, physical activity, relationships with classmates, year of study, and breakfast consumption.

#### Childhood Trauma Questionnaire-Short Form (CTQ-SF)

The questionnaire includes 28 items across five subscales: emotional neglect, physical neglect, physical abuse, sexual abuse and emotional abuse. Each item is scored from 1 to 5 (never = 1, always = 5). Scores for each subscale range from 5 to 25. The total score is the sum of five subscale scores, ranging from 25 to 125. Higher scores indicate more severe childhood trauma. The cut-off values for the presence of each type of traumatic experience are as follows: emotional abuse ≥13, physical abuse ≥10, sexual abuse ≥8, emotional neglect ≥15, and physical neglect ≥10 [10]. In this study, the Cronbach’s α coefficient of the total CTQ-SF scale was 0.60, and the split-half reliability was 0.55.

#### 10-Item Connor-Davidson Resilience Scale (CD-RISC-10) 

The scale contains 10 items to investigate the psychological resilience of college students. Each item is scored from 0 to 4 (almost always = 4, often = 3, sometimes = 2, rarely = 1, never = 0). Lower scores indicate worse psychological resilience. The Cronbach’s α coefficient is 0.86 and the split-half reliability Spearman–Brown coefficient is 0.85 [11].

#### Center for Epidemiologic Studies Depression Scale (CES-D) 

This scale comprises 20 items covering four dimensions: depressive mood, positive mood, physical symptoms, and interpersonal relationships. Each item uses a 4-point rating scale, with lower scores indicating lower levels of depressive symptoms. A total score of <16 indicates the absence of depressive symptoms, whereas a score of ≥16 indicates the presence of depressive symptoms [12, 13].

Based on their CES-D scores, vocational college students were divided into a depressive symptom group (CES-D score ≥16) and a non-depressive symptom group (CES-D score <16).

### Statistical Methods

Data were analysed using SPSS 27.0 (IBM Corp., Armonk, NY, USA) and R software (R Foundation for Statistical Computing, Vienna, Austria). Continuous data were expressed as mean ± standard deviation ($\bar{x}$ ± s). The Shapiro–Wilk test was used to assess normality, and Levene’s test was used to assess homogeneity of variance. As the variances of the CTQ-SF and CD-RISC-10 scores differed significantly between the two groups, Welch’s *t*-test was used for these between-group comparisons. Categorical data were expressed as frequencies and percentages [n (%)], and comparisons between groups were performed using the chi-square test. Multivariable logistic regression analysis was used to analyse the factors associated with depressive symptoms and to assess the multiplicative interaction between CTQ-SF and CD-RISC-10 scores (CTQ-SF score × CD-RISC-10 score). Additive interaction was assessed by the relative excess risk due to interaction (RERI), the attributable proportion due to interaction (AP), and the synergy index (S), and their 95% confidence intervals (CIs) were estimated by the bootstrap method (2000 resamples, seed = 123) in R software. A two-sided *p* < 0.05 was used as the criterion for statistical significance.

## Results

### Prevalence of Depressive Symptoms

Among the 992 vocational college students, the prevalence of depressive symptoms was 19.86% (197/992).

### Comparison of CTQ-SF and CD-RISC-10 Scores Between the Two Groups

The CTQ-SF score of the depressive symptom group was higher than that of the non-depressive group (*p* < 0.001), and the CD-RISC-10 score of the depressive symptom group was lower than that of the non-depressive group (*p* < 0.001), as shown in Table 1.

**Table 1.** **Comparison of the CTQ-SF and CD-RISC-10 scores between the depressive symptom group and the non-depressive group ($\bar{x}$ ± s, score)**.

Group	n	CTQ-SF score	CD-RISC-10 rating	CES-D score
Depressive symptom group	197	52.54 ± 9.86	28.26 ± 5.93	22.41 ± 4.87
Non-depressive group	795	47.25 ± 4.50	32.21 ± 2.65	11.35 ± 3.26
*t*-value		7.344	–9.127	28.634
*p*-value		<0.001	<0.001	<0.001

CTQ-SF, Childhood Trauma Questionnaire-Short Form; CD-RISC-10, 10-item Connor-Davidson Resilience Scale; CES-D, Center for Epidemiologic Studies Depression Scale.

### Comparison of General Information Between the Two Groups

The depressive symptom group was older than the non-depressive symptom group and had higher proportions of students with monthly living expenses <1000 yuan, average or poor relationships with classmates, and divorced or widowed parents (*p* < 0.001), as shown in Table 2.

**Table 2.** **Comparison of general characteristics between the depression symptom group and the non-depression group [$\bar{x}$ ± s, *n* (%)]**.

General information		Depressive symptom group (*n* = 197)	Non-depressed group (*n* = 795)	*t*/χ^2^	*p*-value
Age (years)		19.83 ± 2.35	18.98 ± 2.61	4.176	<0.001
Only children					
	Yes	72 (36.55)	322 (40.50)	0.873	0.35
	No	125 (63.45)	473 (59.50)		
Gender					
	Male	85 (43.15)	330 (41.51)	0.113	0.736
	Female	112 (56.85)	465 (58.49)		
Sexual orientation					
	Homosexuality	34 (17.26)	103 (12.96)	2.458	0.293
	Bisexual tendencies	38 (19.29)	160 (20.13)		
	Heterosexual tendencies	125 (63.45)	532 (66.92)		
Family residence					
	Rural areas	124 (62.94)	478 (60.13)	0.414	0.52
	City	73 (37.06)	317 (39.87)		
Monthly living expenses (yuan)					
	<1000	130 (65.99)	263 (33.08)	70.102	<0.001
	≥1000	67 (34.01)	532 (66.92)		
Daily online time (h)					
	<3	75 (38.07)	316 (39.75)	0.122	0.726
	≥3	122 (61.93)	479 (60.25)		
Father’s education level					
	Junior high school and below	110 (55.84)	425 (53.46)	0.27	0.603
	High school and above	87 (44.16)	370 (46.54)		
Sports activities					
	Frequently participate	56 (28.43)	279 (35.09)	2.847	0.092
	Occasionally or not at all	141 (71.57)	516 (64.91)		
Classmate relationship					
	Good	68 (34.52)	523 (65.79)	62.803	<0.001
	Average or poor	129 (65.48)	272 (34.21)		
Grade					
	Freshman	45 (22.84)	157 (19.75)	1.034	0.596
	Sophomore	67 (34.01)	272 (34.21)		
	Junior	85 (43.15)	366 (46.04)		
Breakfast consumption					
	Eat often	78 (39.59)	370 (46.54)	2.802	0.094
	Eat occasionally or not eat	119 (60.41)	425 (53.46)		
Mother’s education level					
	Junior high school and below	115 (58.38)	438 (55.09)	0.562	0.453
	High school and above	82 (41.62)	357 (44.91)		
Parents’ marital status					
	Normal	145 (73.60)	702 (88.30)	26.163	<0.001
	Divorced or widowed	52 (26.40)	93 (11.70)		

1 USD = 6.7459 CNY.

### Analysis of Factors Associated With Depressive Symptoms Among Vocational College Students

Multicollinearity among the independent variables was assessed using tolerance and variance inflation factor (VIF) values. All variables had VIFs < 2 and tolerance values > 0.8, indicating no evidence of substantial multicollinearity, and could be included in subsequent analyses. The results are shown in Table 3.

**Table 3.** **Multicollinearity diagnosis**.

Variable	*B*	Standard error	Beta	*t*	*p*	Tolerance	VIF
CTQ-SF score	0.022	0.002	0.354	13.739	<0.001	0.917	1.09
CD-RISC-10 score	–0.042	0.003	–0.41	–15.921	<0.001	0.916	1.091
Age	0.014	0.004	0.093	3.729	<0.001	0.982	1.018
Only children	0.024	0.02	0.029	1.178	0.239	0.99	1.01
Gender	0	0.02	–0.001	–0.023	0.982	0.968	1.033
Sexual orientation	–0.014	0.014	–0.025	–1.018	0.309	0.992	1.008
Family residence	–0.004	0.02	–0.005	–0.189	0.85	0.987	1.013
Monthly living expenses	–0.137	0.021	–0.168	–6.665	<0.001	0.955	1.047
Daily online time	0	0.02	0	0.013	0.989	0.988	1.012
Father’s education level	–0.028	0.02	–0.035	–1.414	0.158	0.99	1.01
Sports activities	0.035	0.021	0.042	1.676	0.094	0.986	1.015
Classmate relationship	0.118	0.021	0.145	5.722	<0.001	0.947	1.056
Grade	–0.026	0.013	–0.05	–2.011	0.045	0.982	1.019
Breakfast consumption	0.016	0.02	0.019	0.779	0.436	0.978	1.022
Mother’s education level	–0.018	0.02	–0.023	–0.918	0.359	0.984	1.016
Parents’ marital status	0.127	0.028	0.112	4.492	<0.001	0.976	1.024

VIF, variance inflation factor; CTQ-SF, Childhood Trauma Questionnaire-Short Form; CD-RISC-10, 10-item Connor-Davidson Resilience Scale.

The presence of depressive symptoms among vocational college students was used as the dependent variable (present = 1, absent = 0). Age, only-child status, gender, sexual orientation, family residence, monthly living expenses, daily online time, father’s education level, physical activities, classmate relationship, grade, breakfast consumption, mother’s education level, parents’ marital status, CTQ-SF score, CD-RISC-10 score, and their interaction term between CTQ-SF and CD-RISC-10 scores (CTQ-SF score × CD-RISC-10 score) were selected as independent variables. Multivariable logistic regression analysis showed that age (odds ratio (OR) = 1.179, 95% CI: 1.072–1.295), classmate relationship (OR = 3.197, 95% CI: 2.038–5.015), and parents’ marital status (OR = 3.208, 95% CI: 1.788–5.756) were risk factors for depressive symptoms (*p* < 0.05). In contrast, monthly living expenses (OR = 0.264, 95% CI: 0.168–0.416), sexual orientation (OR = 0.740, 95% CI: 0.551–0.993), and CD-RISC-10 score (OR = 0.306, 95% CI: 0.187–0.502) were protective factors (*p* < 0.05). The interaction term between CTQ-SF scores and CD-RISC-10 scores was statistically significant (OR = 1.017, 95% CI: 1.007–1.027, *p* = 0.001), indicating a statistically significant multiplicative interaction. The results are presented in Table 4.

**Table 4.** **Analysis of the influencing factors of depressive symptoms among vocational college students**.

Variable	*B*	OR	95% CI	*p*
CTQ-SF score	–0.275	0.76	0.561–1.028	0.075
CD-RISC-10 score	–1.184	0.306	0.187–0.502	<0.001
CTQ-SF score × CD-RISC-10 score	0.017	1.017	1.007–1.027	0.001
Age	0.164	1.179	1.072–1.295	<0.001
Only children	0.192	1.211	0.770–1.905	0.406
Gender	–0.197	0.821	0.521–1.294	0.395
Sexual orientation	–0.301	0.74	0.551–0.993	0.045
Family residence	–0.151	0.86	0.543–1.361	0.518
Monthly living expenses	–1.33	0.264	0.168–0.416	<0.001
Daily online time	0.044	1.045	0.663–1.647	0.848
Father’s education level	–0.22	0.802	0.515–1.250	0.33
Sports activities	0.232	1.261	0.787–2.021	0.336
Classmate relationship	1.162	3.197	2.038–5.015	<0.001
Grade	–0.277	0.758	0.572–1.005	0.054
Breakfast consumption	0.074	1.076	0.685–1.691	0.75
Mother’s education level	–0.282	0.754	0.480–1.184	0.22
Parents’ marital status	1.166	3.208	1.788–5.756	<0.001

CTQ-SF, Childhood Trauma Questionnaire-Short Form; CD-RISC-10, 10-item Connor-Davidson Resilience Scale.

### Analysis of the Additive Interaction Between CTQ-SF and CD-RISC-10 Scores on Depressive Symptoms in College Students

With the low trauma + high resilience group (−/−) as the reference, the ORs (95% CIs) of the high trauma + high resilience (+/−), low trauma + low resilience (−/+), and high trauma + low resilience (+/+) groups were 0.427 (0.255–0.714), 1.218 (0.686–2.165), and 0.644 (0.312–1.329), respectively. The additive interaction indices were RERI = −0.001 (95% CI: −1.051 to 0.598), AP = −0.002 (95% CI: −1.652 to 1.043), and S = 1.003 (95% CI: −6.567 to 7.063). RERI = 0, AP = 0, and S = 1 indicated no additive interaction between CTQ-SF score and CD-RISC-10 score in relation to depressive symptoms, as shown in Table 5.

**Table 5.** **Analysis of the interaction between CTQ-SF score and CD-RISC-10 score on depressive symptoms in college students**.

Index	Estimate	95% CI	*p*
OR (+/+ vs –/–)	0.644	0.312–1.329	0.234
OR (–/+ vs –/–)	1.218	0.686–2.165	0.501
OR (+/– vs –/–)	0.427	0.255–0.714	0.001
RERI	–0.001	–1.051–0.598	–
AP	–0.002	–1.652–1.043	–
S	1.003	–6.567–7.063	–

RERI, relative excess risk due to interaction; AP, attributable proportion due to interaction; S, synergy index; CTQ-SF, Childhood Trauma Questionnaire-Short Form; CD-RISC-10, 10-item Connor-Davidson Resilience Scale.

## Discussion

The present study showed that 19.86% of the 992 vocational college students had depressive symptoms. Luo *et al*. [14] investigated more than 140,000 Chinese college students during the normalisation of epidemic prevention and control and reported a relatively high prevalence of depressive symptoms, which was associated with various sociodemographic and psychosocial factors. Its research background and sample size provided an important reference for the present study. Wang *et al*. [15] studied the relationships among childhood trauma, psychological resilience and depressive symptoms in Chinese college students and reported a prevalence of depressive symptoms comparable to that observed in the present study. In contrast, another study [16] reported an overall depression prevalence of 28.40% among Chinese college students, which was higher than that observed in the present study. These differences may be attributable to variations in the cut-off value of depressive symptoms, the regional differences in depression prevalence, the use of different scales, and the differences in demographic characteristics of the study populations.

In this study, the depressive symptom group was older than the non-depressive symptom group and had higher proportions of students with low monthly living expenses, average or poor relationships with classmates, and divorced or widowed parents; CD-RISC-10 score was a protective factor for depressive symptoms in vocational college students. Although age was positively correlated with depressive symptoms, the overall age of the students in this study is similar and homogeneous; and as a demographic data, age was not included in the discussion and analysis.

Childhood trauma and parents’ marital status represent relatively uncontrollable factors. Childhood trauma is irreversible, however, it can be intervened by identifying high-risk groups through early screening, but not by direct intervention. Similarly, parental divorce or widowhood is an objective social background and is unchangeable, but its potential psychological impact may be alleviated through supportive measures. Chang *et al*. [17] reported a positive association between childhood trauma and depressive symptoms in college students, which is consistent with the findings of present study. Previous studies [18] have found that childhood trauma can have persistent effects on personal mental health, causing patients to retain childhood trauma memories in adolescence or adulthood, increasing the risk of depressive symptoms. Early adversity can lead to the formation of negative cognitive schemas in individuals through neurobiological mechanisms. Individuals are very likely to focus on negative information and overprocess it, which can lead to negative rumination response patterns, thereby reducing their ability to cope with stressful events and increasing the risk of depression [19]. Previous reports [20] have pointed out that individuals with childhood trauma have a significantly higher risk of depression compared with those without such experiences. Children from divorced families may experience difficulties adapting to changes in family relationships. This group is extremely distressed when faced with the departure or loss of their father or mother, and their mood remains low, depressed, and pessimistic.

In addition, current peer relationships and social support, as well as CD-RISC-10 scores, may represent potentially modifiable factors. Peer relationships can be improved through campus interventions, such as interpersonal skills training and establishing peer support groups. A previous study has shown that college students with poorer peer relationships had a higher incidence of mental illnesses such as depression, which is consistent with the findings in the present study [21]. Most college students have strong personalities and prominent self-awareness. They are not good at handling interpersonal relationships. In the shared living and learning environment, various conflicts may occur. If such conflicts are not handled properly, they are prone to cause unpleasant emotions and adversely affect the mental health of college students.

Wang *et al*. [15] showed that psychological resilience is negatively correlated with depressive symptoms of college students, which is consistent with the findings of the present study. In the present study, CD-RISC-10 is a protective factor of depressive symptoms. Psychological resilience can be improved through systematic training. College students with higher levels of psychological resilience have more positive cognitive thinking. They can actively cope with and adapt to difficulties, thereby reducing their susceptibility to depression. In addition, living expenses may reflect the extent of students’ financial pressure. Students with limited financial resources may have fewer opportunities for social activities and may experience difficulties in purchasing teaching materials, which could be associated with lower self-efficacy and greater negative emotions in the long run.

The innovation of this study lies in further analysing the interaction between CTQ-SF and CD-RISC-10 scores in relation to depressive symptoms among college students. The additive interaction analysis yielded RERI = −0.001 (95% CI: −1.051 to 0.598), AP = –0.002 (95% CI: −1.652 to 1.043), and S = 1.003 (95% CI: −6.567 to 7.063). Because the 95% CIs for RERI and AP included 0 and that for S included 1, no additive interaction was observed between the CTQ-SF score and the CD-RISC-10 score on depressive symptoms in college students. These findings should be further validated through large-scale studies. With the low trauma + high resilience (−/−) group as the reference, the OR of the high trauma + high resilience (+/−) group (0.427) was lower than that of the high trauma + low resilience (+/+) group (0.644), which is consistent with the protective role of psychological resilience. However, the 95% CI for the high trauma + low resilience group included 1, suggesting no statistically significant difference, which may be attributable to sampling error.

The two scales were used together upon freshman enrolment to identify a “double vulnerable” group with high CTQ-SF scores and low CD-RISC-10 scores. These individuals could be prioritised for psychological support to reduce the risk of depressive symptoms. Specialised psychological assessments could also be considered for students from divorced/widowed families and those facing financial difficulties, with follow-up records established to facilitate ongoing support. Furthermore, study groups or living groups could be formed to create opportunities for peer interaction and social support, taking into account factors such as peer relationships and living expenses. For students experiencing financial difficulties, appropriate financial assistance or subsidies could be provided by the school according to their individual needs.

This study has certain limitations. First, it was conducted at a single-centre and included a limited number of vocational colleges, which may limit the generalisability of the findings to other populations and settings. Second, the CTQ-SF demonstrated relatively low reliability and internal consistency bias, which may have introduced measurement error. Such measurement error may result in attenuation bias and lead to an underestimation of the association between childhood trauma and depressive symptoms and their potential interaction effect. Future studies could include participants from a larger number of vocational colleges and conduct multi-centre studies to further analyse the relationship among psychological resilience, childhood trauma, and depressive symptoms in vocational college students.

## Conclusions

No statistically significant additive interaction was observed between CTQ-SF and CD-RISC-10 scores in relation to depressive symptoms in vocational college students. Colleges and universities should consider monitoring childhood trauma and psychological resilience among students and providing appropriate psychological support for those who may be at increased risk of depressive symptoms.

## Availability of Data and Materials

The datasets used and/or analysed during the current study were available from the corresponding author on reasonable request.
